# Ailing neurosurgical services in rural Africa

**DOI:** 10.1097/JS9.0000000000000020

**Published:** 2023-03-03

**Authors:** Wireko A. Awuah, Favour T. Adebusoye, Pearl O. Tenkorang, Aashna Mehta, Mubarak J. Mustapha, Anastasia F. Debrah, Aymar Akilimali, Jyi Cheng Ng, Toufik Abdul-Rahman, Vladyslav Sikora

**Affiliations:** a Sumy State University, Sumy, Ukraine; b University of Ghana Medical School, Accra, Ghana; c University of Debrecen, Faculty of Medicine, Debrecen, Hungary; d Faculty of Basic Medical Sciences, University of Ilorin, Ilorin, Nigeria; e Faculty of Medicine, Official University of Bukavu, Bukavu, DR Congo; f Faculty of Medicine and Health Sciences, University of Putra Malaysia, Serdang, Malaysia

## Introduction

Inequitable access to neurosurgical services has been a major global issue for decades. This issue has impacted most of the world’s population, particularly those living in low-income communities^1^. Unsurprisingly, rural areas in the poorest regions have been the most underserved in terms of neurosurgical access.

Approximately five billion people do not have access to safe, inexpensive surgical, and anesthetic treatment. Access is most limited in low- and middle-income countries, where nine out of ten people lack access to basic surgical treatment^2^. Every year, low- and middle-income countries require 143 million more surgical procedures to save lives and prevent disability. Only 6% of the 313 million operations performed worldwide each year take place in the world’s poorest nations, which account for more than a third of the global population^2^. The poorest regions, particularly Africa, have the highest unmet demand.

Tragically, an estimated 6.8 million individuals passed away in 2009 as a consequence of neurological illnesses^1^. The global economic cost of neurological diseases is expected to reach ~12.3 trillion US dollars between 2015 and 2030. Africa has the fewest neurosurgeons in the world, with an estimated one neurosurgeon for every four million people^3^. The significant shortage of neurosurgeons and healthcare facilities has resulted in extremely inequitable access to neurosurgical care, particularly in rural areas. Rural areas are home to nearly two-thirds of Africans. With such a large deficit in African neurosurgical services, it is evident that the population in these rural areas does not even have access to neurosurgical services.

## Efforts to improve rural neurosurgical access in Africa

Significant efforts have been made to address inequitable neurosurgical access in Africa, particularly in rural areas. Innovative and rapid training programs have increased the rate of mastery of common neurosurgical operations, and technological advances have been used to bridge communications and triage across large distances, resulting in improved rural outcomes that are frequently equivalent to metropolitan care^1^.

The Fellowship of the College of Surgeons in Neurosurgery (FCS-ecsa-NS) was established with the goal of training local neurosurgeons to improve regional and rural neurosurgical access in Africa. Despite significant investments and efforts to run such a rigorous fellowship, the rural population has yet to see improvements to their problems^4^. Meanwhile, Tanzania made significant strides in rural neurosurgical services when a local hospital decided to offer non-neurosurgeons a 6-month intensive neurosurgery course. They were trained to perform simple neurosurgical procedures, which resulted in an increase from 18 procedures in 2005 to 92 procedures per year from 2008 to 2010^1^. This begs the serious question of how to entice non-neurosurgeons to pursue neurosurgical training. Given the total lack of access to neurosurgeons in these rural communities, this initiative appears to be a good idea for most African healthcare systems. Over 47 African countries have embraced this concept, especially since non-neurosurgeons can successfully perform these simple procedures^5^. The serious question is, what about complex neurosurgical cases that are beyond the knowledge and expertise of these non-neurosurgeons?

Furthermore, the Duke Medical Center collaborated with Ugandan Health facilities to establish a training program with the goal of increasing Uganda’s neurosurgical capacity, particularly in rural areas^6^. Duke Medical Center provided 1400 pieces of equipment, including microscopes, anesthesia machines, and monitoring equipment, as part of this program^6^. This incredible initiative resulted in a 313% increase in neurosurgeon capacity^6^. Despite the fact that such programs are extremely beneficial and have produced positive results, insufficient funding, inadequate equipment, and logistical challenges continue to stymie progress in neurosurgical care in African rural areas^6^.

## Rural neurosurgical service gaps in Africa

Rural neurosurgery continues to face significant challenges across Africa and even on a global scale. The primary challenges include a lopsided urbanization of neurosurgeons, a lack of access to cutting-edge technology, and lengthy travel times between rural populations and neurosurgical qualified facilities^1^. There is no reliable source of equipment or logistics in these rural areas, stifling progress in neurosurgical care in Rural Africa^1^. For instance, In Mulago hospital, a rural Ugandan hospital, the staff in the neurosurgical operating room cannot perform more than two surgeries per day due to a lack of functioning sterilization machines^6^.

One of Africa’s most pressing problems is a severe shortage of physicians and neurosurgeons. Despite the fact that these African countries have made significant progress in bridging neurosurgeon deficits in rural areas by training nonphysicians to perform simple neurosurgical procedures, the importance of well-trained physicians and neurosurgical specialists cannot be overstated. Most African healthcare workers migrate from impoverished countries to more developed countries, resulting in a severe shortage of healthcare providers in areas where the need is greatest. Financial reasons, limited career options, interpersonal conflicts among inconsistently qualified professionals, poor working conditions, and political instabilities are among the push factors driving this qualified physician migration^7^. Nigeria, Africa’s most populous country, has only 27 neurosurgeons serving an enormous population of over 170 million people^3^. Also, the Democratic Republic of Congo (DRC)’s has only 16 local dwelling neurosurgeons for a population of 95 million people which has an abysmal ratio of 1 neurosurgeon to 5.9 million Congolese population^8^.

Ghana, another Sub-Saharan African country with a population of 31 million, has only 25 neurosurgeons^9^. There is a huge disparity in access to neurosurgical services in Ghana, with neurosurgical services available in only four of the 16 regions. This means that more than 75 percent of Ghana’s population lacks total neurosurgery access, and there is no guarantee that the remaining 25 percent has equitable and up-to-date access^9^.

In some instances where there are at least neurosurgical services, patients will have to wait for several weeks to even months to get access to neurosurgical care. An example is the neurosurgery department at Bugando Medical Facility (BMC), a tertiary referral center in Tanzania where a number of patients will have to wait several weeks and months to get surgical care^7^. BMC technically serves a region of nearly 13 million people, however there is surprisingly only one licensed local neurosurgeon and two licensed local anesthesiologists serving this huge population. Elective surgery is performed just once each week^7^.

Cost, poor distribution of medications, inadequate power supplies, lack of advanced surgical equipment, sanitation, unavailability of primary physician care, and education about advanced specialty care are some of the main barriers to successful regional and rural neurosurgical care in Africa, particularly Liberia^10^. In advanced countries, the mortality associated with these minor issues is usually avoidable. This is due to people having easier access to basic healthcare, as well as greater medical knowledge and the ability to follow up with patients on a regular basis. In almost all healthcare settings, basic primary care, in addition to perioperative and postoperative care, is required. Given the importance of patient care, performing neurosurgery or other specialty therapy with limited equipment puts surgeons in a difficult position to assist patients appropriately^10^.

To make matters worse, it is difficult to track progress in neurosurgical procedures because most studies to assess progress in neurosurgical care in rural Africa are not properly conducted^1^. This simply means that studies are limited, and sample sizes are small, resulting in an underestimation of the actual problems that rural Africa faces in terms of neurosurgical care^1^.

## Recommendations

It is necessary to establish initiatives to augment neurosurgical capacity and strengthen health systems in order to address the challenges faced in addressing the local burden of neurosurgical disease. In the foreseeable future, it is doubtful that any African neurosurgery facility will have all the essential resources. Schemes to raise public awareness about the repercussions of restricted neurosurgical services in rural regions should be developed.

The major challenges that rural healthcare faces, particularly in Africa, are massive healthcare infrastructure deficits^11^. As a result, massive investments in infrastructure for healthcare services in rural areas are required, such as the construction of more rural hospitals with government or external support. It is well understood that neurosurgery is an expensive field to invest in, but strategies for providing low-cost neurosurgical care at the regional and rural levels should be developed. Furthermore, government intervention in the form of funding reimbursement to facilitate intrahospital equipment such as stereotactic systems, trinocular microscopes, neuroendoscopes, and so on, as well as extrahospital facilities such as dependable solar power to provide sustainable neurosurgical services in rural areas should be executed. Proper planning and design of the site of neurosurgical facilities surrounding rural regions, while taking into account unavailable quality infrastructures such as substandard roads, is highly recommended in order to make surgical treatments easily accessible.

The majority of neurosurgical cases in impoverished areas can be avoided by taking appropriate preventative measures. Expanding global health outreach programs, particularly in rural areas, is critical for these preventive measures. It will be extremely beneficial to consistently raise awareness of the importance of taking the necessary precautions to prevent certain neurological diseases. For example, advocating for and providing low-cost screening services for pregnant women to reduce the prevalence of a number of congenital central nervous system disorders. There could also be well-planned strategies to avoid the most common neurosurgical cases, such as traumatic brain injuries. Traumatic brain injury, which is typically caused by a road accident, accounts for the majority of neurosurgical cases in African rural communities and could be avoided by educating the rural population about road safety precautions.

Nonetheless, innovations such as telehealth, mobile neurosurgical practices, and refresher courses for life-saving operations should be implemented. To address major challenges in rural neurosurgical practices, it may be necessary to develop specific guidelines for group collaborations with both rural clinics and college teaching hospitals, to provide surplus technological innovation to rural areas, and to quickly educate rural surgical staff members. These actions could result in long-term feed-forward initiatives for trainees as well as infrastructural solutions, promoting rural health and making it comparable to urbanized care.

Authorities in African nations should try innovative strategies to train more healthcare physicians while also drastically reducing healthcare worker emigration. Some of these strategies include improving working conditions for health professionals, providing good training and excellent career opportunities, good salary and mandatory service for set periods of time. Additionally, approaches to improve data collection and storage are required to improve job flow effectiveness and allow hospitals to self-evaluate and make adjustments. Furthermore, official triage criteria and reference policies should exist at both the local clinic level and the government department of health in order to develop a better and more effective plan that will ensure that every patient has access to timely healthcare services.

Finally, better investments in rural research are needed to fully understand the needs of these impoverished communities. The true needs of these poor populations are completely unknown.

## Ethical approval and consent to participate

None.

## Sources of funding

None.

## Authors’ contributions

W.A.A.: conceptualization ideas. All authors contributed to the data curation, writing the initial draft, reviewing and editing, and approval of final manuscript.

## Conflicts of interest disclosure

The authors declare that they have no financial conflict of interest with regard to the content of this report.

## Research registration unique identifying number (UIN)

None.

## Guarantor

Aymar Akilimali.

## Consent for publication

None.

## Competing interests

None.

## Availability of supporting data

No new data generated.
